# COVID‐19: The immediate response of european academic dental institutions and future implications for dental education

**DOI:** 10.1111/eje.12542

**Published:** 2020-07-13

**Authors:** Barry Quinn, James Field, Ronald Gorter, Ilze Akota, Maria‐Cristina Manzanares, Corrado Paganelli, Julia Davies, Jonathan Dixon, Gerber Gabor, Rui Amaral Mendes, Petra Hahn, Sibylle Vital, Judith O’Brien, Denis Murphy, Stéphanie Tubert‐Jeannin

**Affiliations:** ^1^ King’s College London, Faculty of Dentistry, Oral and Craniofacial Sciences London UK; ^2^ Cardiff University Sheffield UK; ^3^ Academisch Centrum Tandheelkunde Amsterdam Amsterdam Netherlands; ^4^ Riga Stradins University Riga Latvia; ^5^ University of Barcelona Barcelona Spain; ^6^ University of Brescia Brescia Italy; ^7^ Malmo University Malmo Sweden; ^8^ Semmelweis University Budapest Hungary; ^9^ Centre for Research in Higher Education Policies University of Porto and Case Western Reserve University Cleveland, OH USA; ^10^ University of Freiburg Freiburg Germany; ^11^ University of Paris Paris France; ^12^ Dental Education in Europe Dublin Ireland; ^13^ University Clermont Auvergne Clermont‐Ferrand France

**Keywords:** COVID‐19, dental education, impact, public health, survey

## Abstract

The COVID‐19 pandemic has had an immediate and dramatic impact on dental education. The Association of Dental Education in Europe decided to carry out an investigation to assess the immediate response of European Academic Dental Institutions. An online survey was sent to both member and non‐member dental schools to investigate the impact on non‐clinical and clinical education, assessment and the well‐being/pastoral care measures implemented. The preliminary findings and discussion are presented in this paper, for the responses collected between the 25 March and 5 April 2020. The survey at this time of publication is ongoing, and detailed results can be accessed https://adee.org/covid‐19‐european‐dental‐education%E2%80%99s‐immediate‐response.

## 1 INTRODUCTION

In December 2019, several patients in Wuhan, China presented with pneumonia of unknown aetiology. On 8 January 2020, the causative agent was announced as a novel coronavirus.^1^ The World Health Organisation (WHO) on 30 January 2020 announced that this outbreak constituted a public health emergency of international concern.^2^ The transmission of this virus is associated with human‐to‐human contact via droplets and aerosols.^3^ On the 15 March 2020, many European countries began to implement a lockdown of their population and dental offices were asked to close. Furthermore, dentists were reported to have one of the highest professional risks of being infected by coronavirus and a possible vector of transmission.^4^

As the pandemic spread, the experiences of oral health professions in clinical practice varied considerably depending on national context. Due to the rapid spread of COVID‐19 across Europe in the first quarter of 2020, it was essential for dental academic institutions to make appropriate and timely modifications to the delivery of education and clinical care, in order to protect patients, students and staff but also sustain continued student academic progress.

The Association of Dental Education in Europe (ADEE) has always advocated for high quality dental education, as well as for the central role that Oral Health Professionals can have in public health. ADEE therefore felt immediately involved in the dynamic of the collective effort required of all and became aware of the strong need for collaboration and information exchange at the European level. ADEE was at the heart of discussions with our member institutions and wider international sister organisations and decided to carry out a preliminary investigation into the immediate response of European Academic Dental Institutions into the COVID‐19 pandemic. Given the changing COVID‐19 situation and the fact that the survey is still ongoing, the authors chose to present the initial results in a commentary that will be followed later by an original paper.

## 2 METHODS

The aim of this questionnaire survey was to get the first picture of the initial response of European dental schools to the COVID‐19 crisis and determine what was informing their decisions at this stage. The purpose of this survey is also to create a map of the responding faculties, to enable the building of a community of dental education institutions in Europe.

An electronic ad hoc questionnaire was developed by consensus with the ADEE executive and O‐Health‐Edu Erasmus plus partners and was piloted by colleagues at the executives’ associated dental schools. The questionnaire used simple single‐answer or multiple‐choice questions to assess clinical and non‐clinical teaching, examinations and well‐being/pastoral care of students and staff as well as opinions about the future of dental education and role of oral health workforces. The issues related to availability and use of PPE was not explored in the questionnaire.

It was distributed to ADEE member institutions and sent to French dentals’ schools through an academic thematic group. The survey launched on the 25 March 2020 to 153 dental schools.

The data collected were analysed with descriptive statistics and can be accessed on the ADEE website: https://adee.org/covid‐19‐european‐dental‐education%E2%80%99s‐immediate‐response. This commentary reports the first results obtained as of 5 April 2020, but the survey is still ongoing, and its content is likely to evolve as the pandemic and its consequences for dental education evolve. The online map and charts report real‐time data collection.

## 3 RESULTS

The initial responses, recounted here, were collected from the 25th of March to the 5th of April 2020. During that period 69 of the 153 schools responded. The survey at the time of publication remains open for further data collection. The reported data cover five main areas: clinical activities, non‐clinical teaching; assessment; pastoral support and future implications.

### 3.1 Clinical‐based education

Schools reported that activities permitted in the university dental clinics were very limited, with access mainly permitted for managing only emergency dental treatments or urgent, non‐delayable dental treatments and with a preference to provide dental care for vulnerable patients. In dental hospitals, the clinical work was mainly being performed by the senior staff (96%) with some participation of postgraduate students (30%) with undergraduate students being asked to help only in non‐clinical activities (11%). Concerning the defining of new protocols for dental care within the COVID‐19 context, dental academics said that they relied on a mix of knowledge sources, such dental literature or local standardised protocols for infectious diseases but the majority were mainly following national guidelines or directives.

### 3.2 Non‐clinical teaching

All the schools that reported restrictions of access to academic buildings planned to provide or were already providing online teaching to replace the planned taught material. Ninety per cent of schools reported using online pedagogical software tools, with 72% using live or streamed videos, links to other online materials (48%), organisation of virtual meetings (65%) and also less frequently small scale working groups, social media groups or journal clubs.

### 3.3 Assessment

The lockdown has led dental schools to postpone formative (46%) and summative assessments (42%), or to organise examinations entirely online (50%). Some schools were aspiring still to have some examination elements held in person (19%). Most schools (72%) were considering postponing the evaluation of required clinical competences for the students. They also were planning to change their assessment schedule or extend program dates, particularly with regard to clinical hours rather than reduce the clinical requirements in order to graduate.

### 3.4 Well‐being and Pastoral support

Concerning the management of student and academic staff stress, it appeared that almost 30% of the responding schools had no COVID19 specific support, where this support existed it was mainly managed centrally by the university (circa 50%). The types of support provided were mainly for students or staff being provided with an academic contact or emergency phone number in case of difficulty. Dedicated web pages for support or discussion via online meetings were less often offered to students.

### 3.5 Future implications

For the schools which replied, responses up to 5 April 2020 suggested that the COVID‐19 crisis will change the way in which public health education plays a role in dental education (90%) and also the shape of the oral health workforce (66%). At this stage of the survey, schools were unsure what would be the long‐term impact on clinical dental education.

## 4 DISCUSSION

This preliminary survey gives an insight into European academic dental institutions’ immediate response to the COVID‐19 crisis from 25 March to 5 April 2020. Given the speed of arrival of the COVID‐19 pandemic, the dental academic institutions across Europe have responded in agile and innovative ways to ensure the continued education of the future dental workforce. The issue of clinical competence assessment is, of course, an ongoing concern. Nevertheless, practical solutions appropriate to the existing resources of the institutes, countries and individuals involved are being found, such as clinical reasoning exercises, case presentation narratives and self‐reflection activities based on portfolios/log diaries.

First, it must be stated that dental education is different from medical education, in that a high proportion of the dental clinical education requires that the students convene in physical settings and cannot be replaced by telehealth formats, which are now occurring for our medical colleagues.^5^ Many schools intend to use the present period of “lockdown,” to front load the curriculum with academic activities involving online learning, in the hope that the students will have more time on the clinics when they return. Similarly, examinations are being transitioned to online formats when previously final examinations were considered off‐limits as far as online innovation was concerned.^6^

The impact on students’ and staff wellbeing may result in increased stress, due to the increased volume of online meetings, emails and demands to be “ever‐present” as individuals struggle with establishing boundaries in the new work‐life realities.^7^

The challenges of managing, for a sustained and undefined period, no face‐to‐face education or clinical patient chairside activities, are therefore an unprecedented task that calls into question the foundations and the future of dental education. The immediate impact of this pandemic has been recorded by this survey but what will happen in the medium‐to‐long term remains an unknown. The future is uncertain as we do not know when this pandemic will be over, and when and how we will be able to return to a “new normality.” Across Europe customised decisions will need to consider each institution's/country's unique challenges and demands, but at the same time international and especially European cooperation will be indispensable. Given the immediate demonstrated impact on clinical education, it is clear that this crisis is going to significantly affect dental education in the medium‐ and long term. Dental education historically is very “hands‐on” and requires a very high staff to student ratio on the teaching clinics.^8^ Safety is paramount for our patients, students and staff. The COVID‐19 pandemic will lead to reconsidering many aspects of our clinical teaching including, arrangement of dental clinics, control of aerosols and airflow, increasing the time to decontaminate surgeries and reconsidering what is appropriate personal protective equipment. It may also impact the appeal of the dental care professions to students, at least in the immediate future.

Building on these initial results, the ADEE executive considers the likely mid‐ and long‐term responses and implications of the COVID‐19 pandemic will no doubt vary with each country's unique challenges and demands, but generally the following points should be considered. Until a vaccine is developed, social distancing in some form will have to be in place. Thus, depending on how long students and staff are excluded from clinics, the academic timetable may require changes to cover non‐clinical elements at the beginning of the forthcoming academic year. Teaching and assessments are now being provided online. E‐learning had been increasing in dental education along with blended educational techniques before the COVID‐19 pandemic.^9^ We can only speculate how will this be taken forward in the future; will this continue, or will there be a shift back to traditional methods? This in turn will have an impact on all the activities carried out in dental schools. In the clinical setting, many questions will also arise relating to the validity of antibody tests for determining how and by whom dental clinical care can be accessed ^10^ or to the availability of sufficient and adequate quality personal protective equipment (PPE). Indeed, what will be the necessary standard of PPE, post the COVID‐19 pandemic? We already know that reorganisation of clinics will be needed with students/ staff attending on a rota basis with longer time allocations for patient appointments. The infrastructure of clinics may also need to be changed in the longer term with regard to the spacing and isolation of dental units and air‐conditioning. In that context, pastoral support for students and staff will be vital to help them cope with the fall‐out from this current crisis. There are of course also the associated financial implications and who will pay for such changes?

The COVID‐19 pandemic will have lasting impact on dental education but it may also change the shape of the oral health workforce. Public health education will perhaps take a more prominent role in the education of our workforce. This COVID‐19 pandemic has resulted in a paradigm shift in our education and future clinical provision. In some countries at least, the oral healthcare team has been redeployed to assist in the care of COVID‐19 patients working in inter‐disciplinary teams.^11^ In these contexts, the value of our oral healthcare teams as seen by our general health colleagues may encourage greater interprofessional education when we return to our campuses.^12^ Collaborative care may encourage the wider healthcare workforce to include oral healthcare to deliver better community care.^13^ This pandemic will affect many and we must be prepared for future emerging respiratory diseases. We must learn from this experience and remember the binding professional responsibility given by Hippocrates in “Of the Epidemics” is *“primum non nocere”* or *“first do no harm,”* to either our patients, students or staff.^14^ We need to look after each other in these challenging times.

This current study showed how decision‐makers, educators and clinicians need evidence‐based information to make informed decisions. There is thus a need for ongoing research to monitor developments and hence ADEE will be taking this work forward in an attempt to provide some of the answers to the above. It is important that we do not miss the opportunity to research into how these new methods of engagement with our students and colleagues have impacted the learning environment. A scholarly approach is imperative for reflecting and evaluating the results achieved. ADEE wish to collect further data and analyse what will be the ultimate impact of this pandemic with a follow‐up questionnaire. We will therefore continue to engage with our member schools to collect relevant data, monitor the ongoing situation, share evolving best practice, and communicate our findings to improve our preparedness for future pandemics.
